# Supplementary material for: Simultaneous single-cell three-dimensional genome and gene expression profiling uncovers dynamic enhancer connectivity underlying olfactory receptor choice
Source: Nat Methods. 2024 Apr 15;21(6):974–82. doi: 10.1038/s41592-024-02239-0 (PMC11166570; doi:10.1038/s41592-024-02239-0)
Supplement: Supplementary file 1 — Reporting Summary [file 41592_2024_2239_MOESM1_ESM.pdf]

nature portfolio

Corresponding author(s): Xiaoliang Sunney Xie

Last updated by author(s): Jan 16, 2024

Reporting Summary

Nature Portfolio wishes to improve the reproducibility of the work that we publish. This form provides structure for consistency and transparency in reporting. For further information on Nature Portfolio policies, see our Editorial Policies and the Editorial Policy Checklist.

Statistics

For all statistical analyses, confirm that the following items are present in the figure legend, table legend, main text, or Methods section.

n/a	Confirmed	
☐	☒	The exact sample size (*n*) for each experimental group/condition, given as a discrete number and unit of measurement
☐	☒	A statement on whether measurements were taken from distinct samples or whether the same sample was measured repeatedly
☐	☒	The statistical test(s) used AND whether they are one- or two-sided<br>*Only common tests should be described solely by name; describe more complex techniques in the Methods section.*
☒	☐	A description of all covariates tested
☒	☐	A description of any assumptions or corrections, such as tests of normality and adjustment for multiple comparisons
☐	☒	A full description of the statistical parameters including central tendency (e.g. means) or other basic estimates (e.g. regression coefficient) AND variation (e.g. standard deviation) or associated estimates of uncertainty (e.g. confidence intervals)
☐	☒	For null hypothesis testing, the test statistic (e.g. *F*, *t*, *r*) with confidence intervals, effect sizes, degrees of freedom and *P* value noted<br>*Give P values as exact values whenever suitable.*
☒	☐	For Bayesian analysis, information on the choice of priors and Markov chain Monte Carlo settings
☒	☐	For hierarchical and complex designs, identification of the appropriate level for tests and full reporting of outcomes
☐	☒	Estimates of effect sizes (e.g. Cohen's *d*, Pearson's *r*), indicating how they were calculated

Our web collection on statistics for biologists contains articles on many of the points above.

Software and code

Policy information about availability of computer code

Data collection	No software was used.
Data analysis	10X scRNA-seq data were analyzed with Cell Ranger (v 5.0.1) and Seurat (v 4.0.4).<br>METATAC data were processed with custom scripts (https://github.com/sunneyxielab/METATAC_pipeline) and further analyzed with ArchR (v1.0.2).<br>LiMCA RNA data were analyzed with Seurat (v4.2.0).<br>LiMCA Hi-C data were processed and analyzed with dip-c and hickit (r291) package (https://github.com/tanlongzhi/dip-c, https://github.com/lh3/hickit).<br>3D genome structures were visualized with PyMol (v2.4.0). All plots were generated with matplotlib (v3.7.0) and ggplot2 (v3.3.3).<br>Custom code related to this paper is available at https://github.com/zhang-jiankun/LiMCA.<br>Flow cytometry was analyzed with BD FACSDiva v9.0 Software.<br>Circos plot was generated with R package circlize (v0.4.12).

For manuscripts utilizing custom algorithms or software that are central to the research but not yet described in published literature, software must be made available to editors and reviewers. We strongly encourage code deposition in a community repository (e.g. GitHub). See the Nature Portfolio guidelines for submitting code & software for further information.

Data

Policy information about availability of data

All manuscripts must include a data availability statement. This statement should provide the following information, where applicable:

- Accession codes, unique identifiers, or web links for publicly available datasets
- A description of any restrictions on data availability
- For clinical datasets or third party data, please ensure that the statement adheres to our policy

Raw sequencing data generated in this study has been deposited to the Sequence Read Archive (SRA; https://www.ncbi.nlm.nih.gov/sra) under accession number PRJNA1002315. The processed data generated during this study has been uploaded to the Gene Expression Omnibus under accession number GSE240128.
Cell type of each cluster was annotated manually with the help of Enrichr database (https://doi.org/10.1093/nar/gkw377).
Published MOE Dip-C data was downloaded under GEO accession code GSE121791. Published OSN bulk Hi-C data was downloaded from 4DN database (https://data.4dnucleome.org/).

Human research participants

Policy information about studies involving human research participants and Sex and Gender in Research.

Reporting on sex and gender	Not applicable. There is no human participants involved in this study.
Population characteristics	Not applicable.
Recruitment	Not applicable.
Ethics oversight	Not applicable.

Note that full information on the approval of the study protocol must also be provided in the manuscript.

Field-specific reporting

Please select the one below that is the best fit for your research. If you are not sure, read the appropriate sections before making your selection.

☒ Life sciences ☐ Behavioural & social sciences ☐ Ecological, evolutionary & environmental sciences

For a reference copy of the document with all sections, see nature.com/documents/nr-reporting-summary-flat.pdf

Life sciences study design

All studies must disclose on these points even when the disclosure is negative.

Sample size	Sample size was not predetermined. For single-cell joint chromatin architecture and gene expression multi-omics data, it consisting of four cell lines, which includes 220 GM12878 cells, 63 K562 cells, 42 eHAP cells and 63 BJ cells, and 411 cells dissociated from the mouse main olfactory epithelium. For single-cell chromatin accessibility data, we profiled 11,880 single cells from the mouse main olfactory epithelium during the first postnatal month. For single-cell RNA-seq data, we collected 73,577 single cells from the mouse main olfactory epithelium during the first postnatal month.
Data exclusions	For the statistic analysis of spatial relationship between expressed ORs and their enhancers in Figure3, several cells were excluded due the unknown allele of expressed ORs of bad quality of 3D structures, as listed in supplementary table 4.
Replication	For single-cell ATAC-seq data, each mouse age was generated with two independent sampling replicates. All attempts at replication were successful.
Randomization	Randomization was not required since our study is based on sequencing. For different group analysis, cells were allocated according to expression level OR the stage of olfactory receptor expression. Random grouping control was down in these analysis to confirm the conclusion.
Blinding	Blinding was not required since our sample is taken from wild-type mice or normal cultured cell line. Since the mice was not genetically engineered and cell line was taken from normal culture with perturbation.

Reporting for specific materials, systems and methods

We require information from authors about some types of materials, experimental systems and methods used in many studies. Here, indicate whether each material, system or method listed is relevant to your study. If you are not sure if a list item applies to your research, read the appropriate section before selecting a response.

Materials & experimental systems

n/a	Involved in the study
☒	☐ Antibodies
☐	☒ Eukaryotic cell lines
☒	☐ Palaeontology and archaeology
☐	☒ Animals and other organisms
☒	☐ Clinical data
☒	☐ Dual use research of concern

Methods

n/a	Involved in the study
☒	☐ ChIP-seq
☐	☒ Flow cytometry
☒	☐ MRI-based neuroimaging

Eukaryotic cell lines

Policy information about cell lines and Sex and Gender in Research

Cell line source(s)	K-562 (ATCC) is derived from the pleural effusion of a 53-year-old female with chronic myelogenous leukemia in terminal blast crises. GM12878 (Coriell Institute) is a EBV-transformed B lymphocyte from a female. BJ (ATCC) cells are fibroblasts established from skin taken from normal foreskin from a neonatal male. eHAP (Cellosaurus) is haploid cell derived from HAP1, HAP1 is a near-haploid human cell line derived from KBM7, a human myeloid leukemia cell line developed from a 39-year-old male.
Authentication	All cell lines were validated with morphology and gene expression and other epigenetic states with published datasets.
Mycoplasma contamination	Mycoplasma contamination test is negative.
Commonly misidentified lines (See ICLAC register)	No commonly misidentified cell lines were used in the study.

Animals and other research organisms

Policy information about studies involving animals; ARRIVE guidelines recommended for reporting animal research, and Sex and Gender in Research

Laboratory animals	Female and male, postnatal day 3-120, CAST/EiJ x C57BL/6J hybrid mice, female and male, postnatal day 1-60, DBA/2J x c57BL/6J hybrid mice.
Wild animals	No wild animals were used.
Reporting on sex	Both female and male mice were used. The conclusion derived from this study is not biased to specific sex.
Field-collected samples	No field-collected samples were used.
Ethics oversight	The study was approved by the Peking University Institutional Animal Care and Use Committee (IACUC). All the animal experiments were conducted following their guidelines.

Note that full information on the approval of the study protocol must also be provided in the manuscript.

Flow Cytometry

Plots

Confirm that:

☒ The axis labels state the marker and fluorochrome used (e.g. CD4-FITC).

☒ The axis scales are clearly visible. Include numbers along axes only for bottom left plot of group (a 'group' is an analysis of identical markers).

☒ All plots are contour plots with outliers or pseudocolor plots.

☒ A numerical value for number of cells or percentage (with statistics) is provided.

Methodology

Sample preparation	The mouse main olfactory epithelium was dissociated into single cell suspension with papain. The single-cell suspension was filtered with 40 um strainer. Then 50000 cells were aliquot to ATAC-seq procedure, briefly, cells were permeabilized and transposed, then stain with 7-AAD.

Instrument	BD, FACS Aria SORP
Software	BD FACSDiva v9.0 Software
Cell population abundance	All nuclei were sorted without biased, the 7-AAD-positive nuclei was selected.
Gating strategy	Nuclei were distinguished from debris based on FSC-A and SSC-A, then the multiplets were removed by two step gating of FSC-W and FSC-H, SSC-W and SSC-H. Then nuclei were selected based on PerCP-cy5-5-A.

☒ Tick this box to confirm that a figure exemplifying the gating strategy is provided in the Supplementary Information.
